# Hybrid System of Emotion Evaluation in Physiotherapeutic Procedures

**DOI:** 10.3390/s20216343

**Published:** 2020-11-06

**Authors:** Patrycja Romaniszyn-Kania, Anita Pollak, Marta Danch-Wierzchowska, Damian Kania, Andrzej P. Myśliwiec, Ewa Piętka, Andrzej W. Mitas

**Affiliations:** 1 Faculty of Biomedical Engineering, Silesian University of Technology, Roosevelta 40, 41-800 Zabrze, Poland; marta.danch-wierzchowska@polsl.pl (M.D.-W.); ewa.pietka@polsl.pl (E.P.); andrzej.mitas@polsl.pl (A.W.M.); 2 Institute of Psychology, University of Silesia in Katowice, Bankowa 12, 40-007 Katowice, Poland; anita.pollak@us.edu.pl; 3 Institute of Physiotherapy and Health Sciences, The Jerzy Kukuczka Academy of Physical Education in Katowice, Mikołowska 72A, 40-065 Katowice, Poland; d.kania@awf.katowice.pl (D.K.); a.mysliwiec@awf.katowice.pl (A.P.M.)

**Keywords:** electrodermal activity, GSR, emotions analysis, psychological analysis, JAWS, clusterisation

## Abstract

Nowadays, the dynamic development of technology allows for the design of systems based on various information sources and their integration into hybrid expert systems. One of the areas of research where such systems are especially helpful is emotion analysis. The sympathetic nervous system controls emotions, while its function is directly reflected by the electrodermal activity (EDA) signal. The presented study aimed to develop a tool and propose a physiological data set to complement the psychological data. The study group consisted of 41 students aged from 19 to 26 years. The presented research protocol was based on the acquisition of the electrodermal activity signal using the Empatica E4 device during three exercises performed in a prototype Disc4Spine system and using the psychological research methods. Different methods (hierarchical and non-hierarchical) of subsequent data clustering and optimisation in the context of emotions experienced were analysed. The best results were obtained for the k-means classifier during Exercise 3 (80.49%) and for the combination of the EDA signal with negative emotions (80.48%). A comparison of accuracy of the k-means classification with the independent division made by a psychologist revealed again the best results for negative emotions (78.05%).

## 1. Introduction

In addition to specific knowledge and qualifications, working with a patient during physiotherapy requires taking care of the communication process and developing positive relationships [1,2]. Emotions experienced during therapy are a reaction of the individual in the form of cognitive evaluation and psychophysiological responses to the physical injury and the effects of treatment achieved in the context of further functioning [3,4]. They are critical for triggering adaptive behaviour that helps to survive and improves the well-being of the individual [5]. Knowledge of emotions allows for a better understanding of coping strategies used in stressful situations and the development of intervention methods aimed at arousing positive emotions, which are a manifestation of health and represent a factor that favours physiological, psychological, and social processes that improve well-being [5,6,7]. Furthermore, identification and interpretation of emotional reactions by means of physiological data acquisition and processing systems can contribute to extending the knowledge of this area [8] and provide an alternative way of data collection. To the best of our knowledge, no research results have been presented to date on the psychophysiological analysis of the patient’s emotional state during rehabilitation. Therefore, the aim of this research is to propose a methodology and to select an optimal set of physiological data to complement the psychological tests and support experts in the evaluation of the patients’ emotional states.

In this paper we propose an objective emotion measurement approach based on the electrodermal activity signal and its comparison with medically approved psychological tests. The signal acquisition setup is integrated with a newly developed rehabilitation installation for physiotherapy of posture defects and scoliosis. Independent and combined evaluations of the results indicate their compatibility and possible implementation in the therapeutic procedure.

## 2. Background

### 2.1. Psychological Assessment

One of the most popular models describing the relationship between stressors and the experienced stress and coping strategies was proposed by Lazarus and Folkman [4]. According to this model, stress is defined as ’a specific relationship between a person and his or her environment which is assessed by a person as aggravating or exceeding his or her resources and threatening their well-being’ [4]. An individual evaluates the elements of the relationship with the environment that are important for his or her well-being and assesses whether the situation represents harm or loss, a threat or a challenge and whether they have sufficient resources to cope with the situation. All these assessments are associated with characteristic emotions [7]. In the case of hurt or loss, the emotions include anger, grief, and sadness, whereas threat is associated with experiencing fear, anxiety, and worry. When stress is assessed as a challenge, the emotional picture becomes more complex and includes both negative and positive emotions such as hope, enthusiasm, excitement, and cheerfulness. Emotions stimulate an individual to be active and lead him or her to seek ways to relieve or endure stress or to find benefits of stressful events [4]. This model is complemented by studies published by Folkman [9] and Folkman and Moscowitz [7] on the importance of positive emotions for coping with stress. A review of studies on the consequences of negative emotions shows that they are harmful to biological health [10] and they can have a healthy effect only in some cases, for example, short-term negative reactions can improve the function of the immune system [11]. Experiencing positive emotions lessens the effects of negative emotions, for example, by reducing physiological arousal [12]. The adaptive role of positive emotions is also manifested in sustaining coping efforts, providing moments of respite to rebuild resources [9]. The latter effect is referred to as extending the available thought-action repertoire and is viewed as a chance to improve mood and health [13]. Furthermore, the Lazarus concept [4] states that the positive or negative emotions should be expected earlier than a conscious or unconscious decision on what kind of emotions an individual experiences and what actions he or she will take [14]. Based on this, the analysis presented in this study focuses on recording physiological changes and analysing negative and positive emotions. Furthermore, two important aspects should also be highlighted. The first aspect concerns the phenomenon of negativity bias, in which negative emotions determine functioning to a greater extent than positive ones, which is related to more intense experiencing of negative emotions [15]. The second aspect refers to the positive balance, which was found not to be a mere balance of positive and negative emotions, but is characterised by the ratio of ca. 3 to 1 [16]. This ratio allows an individual to revive and expand his or her resources and ensures the development and a sense of well-being [13]. An interesting contribution to understanding the importance of positive emotions and the problems discussed is also a study by van Yperen [17], who demonstrated that if positive emotions dominate the emotional picture, the negative ones do not have an effect on the quality of work.

### 2.2. Signal-Based Measures

The autonomic nervous system (ANS) is responsible for emotional stimulation [18]. It is divided into two branches, termed the sympathetic system and parasympathetic system. The direct manifestation of the activity of the sympathetic nervous system responsible for both positive and negative emotions experienced by an individual is the electrodermal activity signal (EDA) [19]. EDA, often commonly called the galvanic skin response (GSR), depends on the activity of sweat glands and the amount of sweat secreted by the eccrine glands, usually in the subcutaneous layer of the hand [20]. The activity of the sympathetic nervous system and changes in skin perspiration are regulated not only by the ambient temperature but also by the central nervous activity related to affective and cognitive states, i.e., emotions [21]. EDA is one of the signals that are most frequently used in the entire field of psychophysiological research because it is capable of reflecting both normal and pathological states [22]. There are two important features of this sympathetic innervation that improve the usefulness of EDA in behavioural medicine [23]. The first feature is that there is no antagonistic parasympathetic innervation of the sweat glands, which means that EDA reflects only sympathetic activity, not sympathovagal balance. The second feature concerns the neurotransmission that mediates the release of acetylcholine. This differs from the noradrenergic neurotransmission typical of other effector sympathetic synapses and additionally makes the EDA signal independent of adrenaline and noradrenaline levels.

The current state of knowledge, which includes the conclusions of research on ANS activity using the available measurement methods, is based, among other things, on the use of sensors fixed on the patient’s chest [24,25]. However, some researchers have shown that it is better to use a wearable wristband as a measuring device for recording physiological signals since patients are accustomed to similar devices and their costs are much lower [26].

The EDA signal has been used in studies to monitor human stress levels. Gjoreski et al. [27] developed an algorithm for the identification of psychological stress in everyday life based on teaching neural networks using laboratory data, thus detecting short-term stress, and a user activity detector that provides contextual data. Different studies have been carried out in Japan on blind and partially sighted people as they moved around in an unknown environment [28]. The EDA signal was correlated with the electroencephalographic (EEG) signal in order to identify factors that increase stress and cognitive strain. The random forest classifier was used to classify a patient’s emotional state as stress or no stress. To detect and understand the mechanism of cognitive load, the electrodermal activity signal was also used [29,30,31]. Many studies have indicated that the level of electrodermal activity correlated with cognitive load and contributed to better diagnosis of this disease. In a study by Vankowa et al. EDA was also indicated as a non-invasive ANS activity biomarker in adolescents affected by Anorexia nervosa (AN), to assess the risk of cardiovascular disorders associated with AN [32].

To the best of our knowledge, no research results have been published to date on the patient’s emotional state during rehabilitation. Studies in the literature have reported only related methods of emotional analysis using external devices and machine learning algorithms. Taheri Nejad and Pollreisz [33] studied the human body’s behaviour when experiencing various types of emotions by recording physiological signals (EDA and heart signals) in a group of 10 university students using the Empatica E4 device. The subjects were presented with videos that were supposed to evoke certain emotions, i.e., happiness, sadness, anger, pain, and fear. One of the key observations was to establish the relationship between EDA and emotions. Furthermore, the number and magnitude of signal peaks turned out to be the most differentiating feature for the emotions studied [33]. Liu et al. [34] proposed another method of the identification of emotions based on the automated selection of features of the EDA signal, using machine learning based on the Support Vector Machine (SVM) algorithm [34]. Emotions were evoked in the study participants in a deliberate way by presenting them with dedicated films. Based on the EDA signal, the authors determined 30 features, which in their opinion led to low accuracy of the classifier. Emotions were also assessed in real time among gamers using EDA and electromyography (EMG) signals to improve game interfaces [35]. In this approach, the EDA signal was correlated with the muscle electrical activity signal and a model based on the Bayesian network used to determine the user’s emotions defined only based on the literature was presented. This proposal is limited by the lack of model validation with feelings experienced by the participants. Another automated method of emotion classification based on wavelet analysis and SVM was proposed by Feng, Golshan and Mahoor [36]. These researchers conducted a study using the EDA signal in 100 children under 30 months old. Each signal received an emotion label assigned by two independent experts, which was then synchronised with the child’s facial expressions and the EDA signal. The results confirmed that the use of wavelet analysis contributes significantly to a more accurate classification of emotions.

## 3. Data Acquisitions

### 3.1. Setup

**Figure 1:**
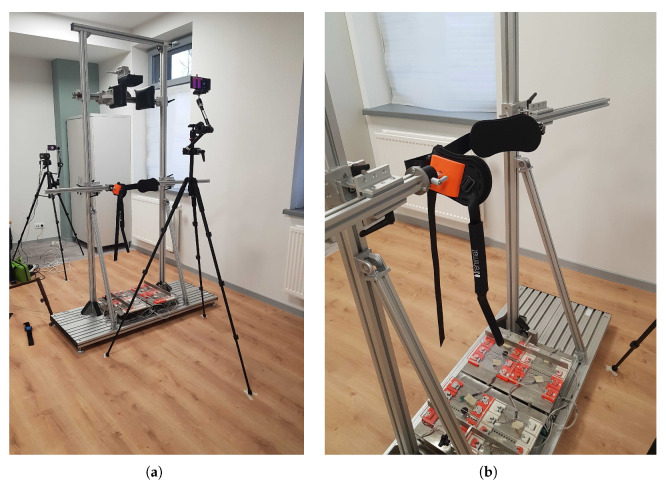
(**a**) A prototype of the D4S module for exercises in the standing position with measuring devices and (**b**) a belt fastened on the patient’s pelvis and a podoscope.

The idea of the Disc4Spine project is to design a comprehensive diagnostic and therapeutic system to support the broadly understood therapy of postural defects and scoliosis. Its basic features include, among others, full diagnostics and continuous monitoring of the therapy, correction of sagittal postural disorders, functional correction during everyday activities or stabilisation of the corrected body posture, and education of patients [37,38]. The system ensures quick and precise detection of postural defects, determination of their complexity, and then helps adjust the therapy to the patient’s needs and continuous monitoring of the defects. The module for exercises in the standing position (Figure 1) is designed as a therapeutic cage for corrective exercises for short single-joint muscles responsible for motor control [39], equipped with measuring devices for the monitoring of the therapy and its progress.

The components of the D4S system include a TOF (Time-of-Flight) camera for the detection of selected anthropometric points and calculation of basic spinal curvatures, two thermal imaging cameras for monitoring temperature changes on the back and face, a device for measuring the distribution of foot pressure on the ground and for measuring the moment of the forces that rotate the lower limb called Rotenso (the platform with red components in Figure 1b). Monitoring of the patient’s physiological functions is possible using the Empatica E4 wristband, which records heart rate variability signals, body temperature and electrodermal activity, allowing for a comprehensive assessment of the patient’s health status before, during and after rehabilitation [40,41]. Furthermore, the cage is equipped with an original system for attaching the belt on the pelvis (orange-red clamp, Figure 1b) allowing for constant monitoring of its angular position in the sagittal, frontal and horizontal planes, which both controls and supports the optimal positioning of the pelvis, whereas during the therapy, it gradually reduces this movement, thus increasing lumbar and clavicular stability. A restraint module which stabilises the head position and influences the range of neck motion is installed over the patient’s head.

### 3.2. Research Protocol

The control group consisted of 41 students (19 women, 22 men) of the Jerzy Kukuczka Academy of Physical Education in Katowice aged from 19 to 26 years (mean 21). The participants voluntarily responded to the invitation to participate in the study, which started from presenting the purpose (analysis of behaviour during rehabilitation), data confidentiality rules, and collecting a written consent for participation in the experiment. The only form of preparation for the experiment was to wear sportswear that ensured freedom of movement. To ensure the study’s high ecological validity, in the research, subjects were asked to perform the exercises according to the given instructions, no other methods of evoking emotions were used except for recreating the situation from a typical rehabilitation session. The exclusion criteria were painfulness during movement tasks, mobility limitations, and lack of understanding of the commands. Inclusion criteria specified that respondents were to:

1. Age in the range 19–29 years (young adults),
2. Be able to provide informed consent for the study,
3. Without any dysfunction in auditory processing, significant visual impairment,
4. Declaring physical activity in the various form of sports exercises,
5. Lack of locomotor dysfunctions that may affect the measurements due to pain or limitations of mobility range,
6. That he/she is not enduring psychiatric treatment and the crisis.

The measurement protocol started with putting on an Empatica E4 wristband. The EDA signal was recorded using the Empatica E4 wristband (E4), which is a medically approved device that allows real-time recording of physiological data. The device’s sampling rate is 4 Hz and provided raw data in .csv format. The wristband is equipped with an electrodermal activity sensor to measure changes in skin electrical conductivity, a photoplethysmographic sensor to measure blood volume pulse from which heart rate variability can be derived, a 3-axis accelerometer to monitor the subject’s activity, and an infrared thermometer. The wristband was always worn on the wrist of the non-dominant limb according to the manufacturer’s guidelines [42].

**Figure 2:**
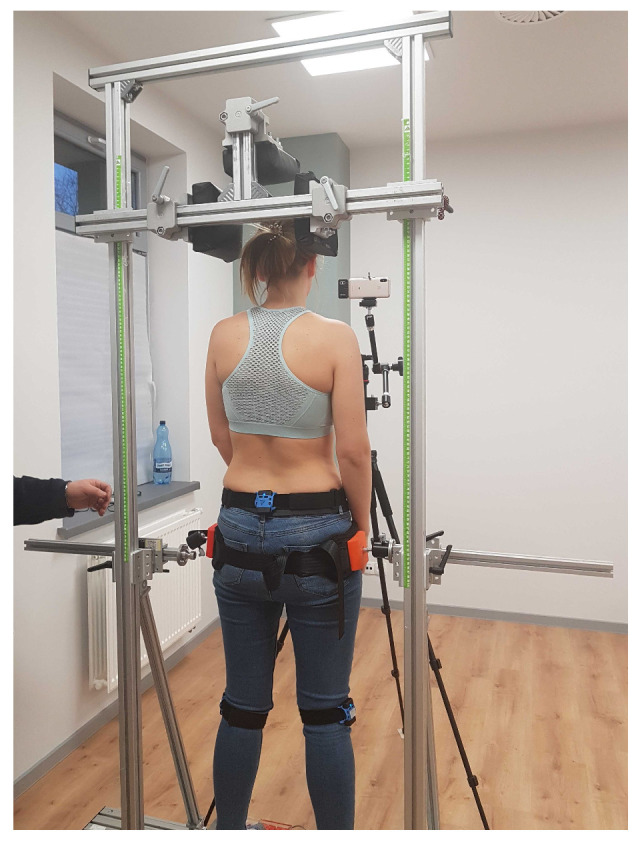
During exercises in the standing position in the D4S module, the head is restrained from two sides to prevent sideways movements; pelvis is restrained by a belt to allow only anterior and posterior tilt.

Next, the participants were placed in a cage for exercises in the standing position (Figure 2). After securing the subject in the system, he or she performed 3 consecutive exercises with different degrees of difficulty depending on the muscles activated:

- Exercise number 1 consisted in performing consecutive anterior and posterior pelvic tilt for 60 s at the frequency of one sequence (anterior/posterior) per second,
- Exercise number 2—the participant was asked to perform the internal rotation of the feet with maximum force for 10 s, during the rotation, each foot was restricted by a beam on the outside,
- Exercise number 3 consisted in performing successively the external rotation of the feet, external rotation of the knees, pelvic anterior tilt, shoulder retraction, and spine elongation. In this position, the participant was asked to remain for 10 s.

The study adopted a declarative approach to the study of emotions. Respondents indicated the emotions they experienced connected with the exercises from among those presented in the questionnaire and what intensity. The questionnaire was in the paper-and-pencil form. Respondents checked the answers immediately after finishing the exercises. The measurement protocol ended with the removal of the E4 wristband.

The Job-related Affective Well-being Scale (JAWS) (short form) was employed in the study to assess emotional reactions caused by physical activity [43]. Since JAWS was originally designed to examine emotions experienced in the working environment, the scale was adapted for the D4S research protocol. The version used in the study consists of 12 items. The items concerned the frequency of particular emotions related to the exercises. A 5-point Likert scale was used (1—never, 5—very often). The reliability of the tool used in the study was calculated using the McDonald’s omega [44]. For the overall result, its value was 0.815, for the subscale of positive emotions −0.850, and for negative emotions −0.71. These results can be considered satisfactory as they exceeded the recommended value of 0.70 [45].

The advantage of JAWS is that the measurement directly concerns the experienced momentary feelings and does not refer to the general beliefs of the participant or his or her attitudes towards a particular activity. Furthermore, the assessment made by the participant is contextual and concerns only the conditions he or she is in at the moment.

## 4. Data Analysis

**Figure 3:**
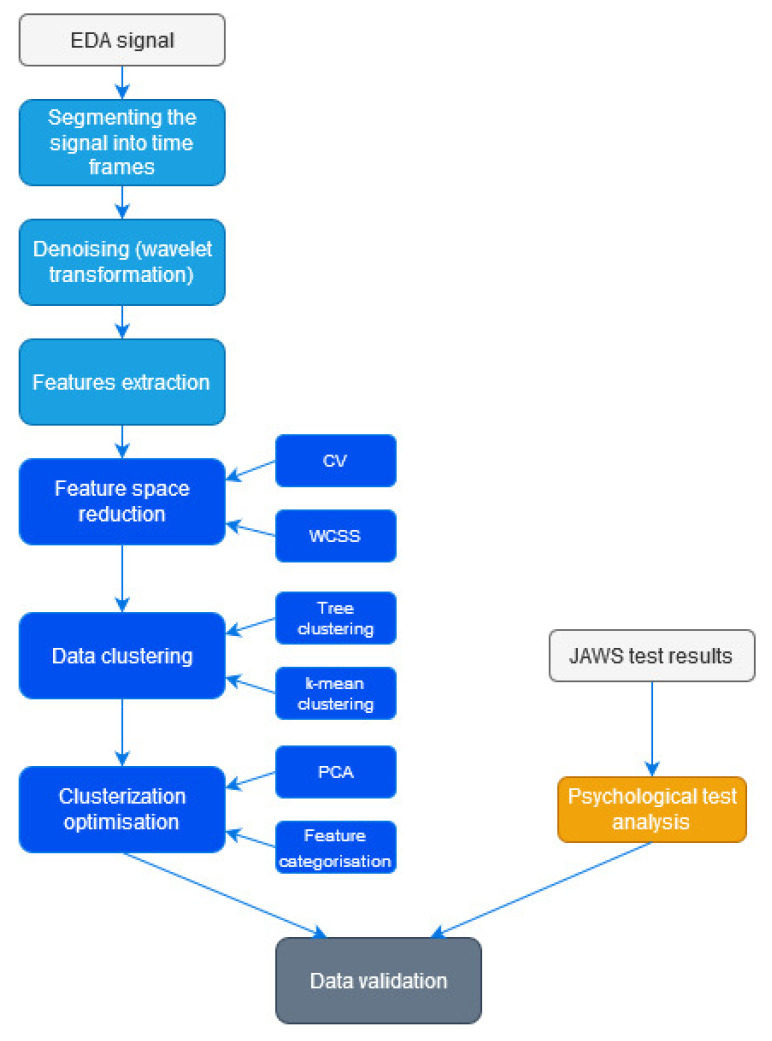
Workflow (grey means the input data, light blue is the preprocessing, dark blue denotes data classification, and yellow is the psychological analysis stage).

The data collected from psychological tests were analysed independently of the EDA measurements by independent experts, i.e., a clinical psychologist and a signal processing specialist. The results were compared in the final phase of analyses (Figure 3).

### 4.1. EDA Signal Preprocessing

**Figure 4:**
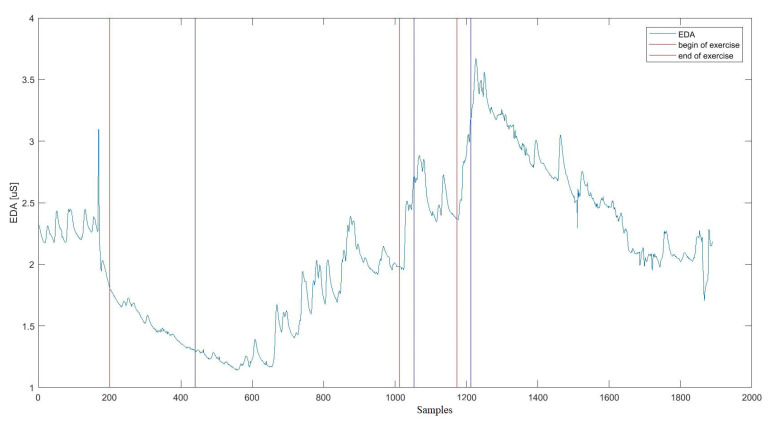
Electrodermal Acvitity (EDA) signal with marked time markers indicating the beginning and end of the exercises: analysed time intervals.

The first step in processing the EDA signal was to divide it into time segments based on time markers marked each time on E4 devices at the beginning of a given activity according to individual stages of the research protocol (Figure 3). For each part of the signal (Figure 4), the following statistical indices were determined: mean, standard deviation, median, variance, 25th and 75th percentiles, quartile deviation, minimum and maximum values during a specific activity, the fourth and fifth-order moments, skewness, kurtosis, root mean square error, total sum, entropy, coefficient of the slope of the regression line allowing to determine the trend-signal tonicity, coefficients of the regression line shift, distance from the values for individual EDA samples from the regression line, number of signal intersections with the regression line. These indices have been emphasised in the literature as important for EDA signal analysis [27,34,46,47,48,49,50].

**Figure 5:**
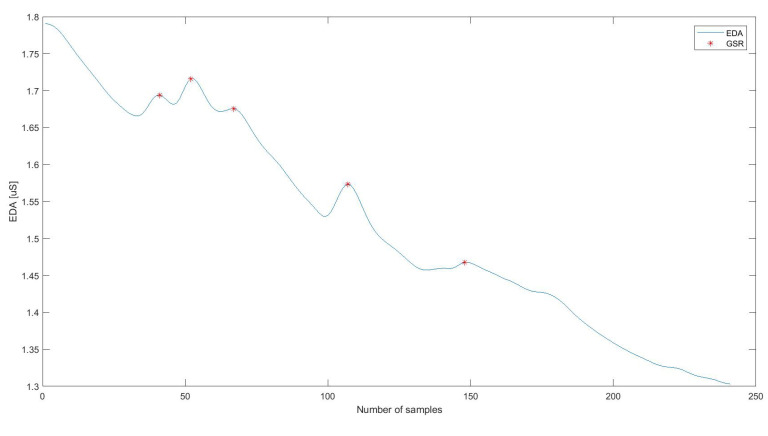
Example of the Electrodermal Activity signal (EDA) with Galvanic Skin Response (GSR).

To denoise the signal, wavelet transform filtration was performed using Symlet wavelet [51], with the maximum level of decomposition set according to Equation (1), and the estimation to obtain a minimum efficiency for mean square error compared to the ideal situation (Figure 5).

(1) $$level=\left\{\begin{array}{cc} log_{2}n & log_{2}n<10\\ 10 & log_{2}n>10 \end{array}\right.$$

The most important set of features for the EDA signal is based on the galvanic skin response (GSR), which is defined as a change in skin electrical resistance as a response to a momentary emotional stimulation, increasing the activity of the sympathetic nervous system [52]. Occurrences of GSR, a sudden change in the EDA signal, is mathematically defined by the local maximum (with a value greater than 0) in the derivative of the phasic component [53] of the signal.

Based on the results, we estimated the following features: rpm—meaning the number of GSRs per minute, the number and energy of GSRs, and the number and energy of significant GSRs (values above 1.5 uS).

### 4.2. Psychological Test Analysis

The results were calculated using two methods [54]. The first method consisted in summing the results for all JAWS questionnaire items after recording the statements about negative emotions. The second method was based on the sum of points for the dimensions of positive emotions (JAWS_pos) and negative emotions (JAWS_neg). The higher the score in the JAWS test, the higher the intensity of emotions experienced during the task performed. The higher the value of the sum for positive emotions, the greater the frequency of positive emotions experienced during the exercises. Similarly, the higher the score in the subsum of negative emotions, the higher the frequency of experiencing negative emotions during the test.

### 4.3. Data Classification

As mentioned before, the research protocol consisted of three different activities. Each step presented below was performed independently, for each stage of the test.

#### 4.3.1. Features Reduction

The features were reduced by using the coefficient of variation (CV) in order to initially determine the most important parameters of the EDA signal [55]. It reflects the degree of variation in the distribution of features and measures the part of the mean value of a feature that is represented by standard deviation. Higher values of this measure mean higher variability of the feature. Unlike standard deviation, the coefficient of variation is a relative measure and is calculated according to the following equation:

(2) $$CV=\frac{\sigma }{\bar{x}}$$

where: σ—standard deviation of the sample, and $\bar{x}$—arithmetic mean of the sample.

**Figure 6:**
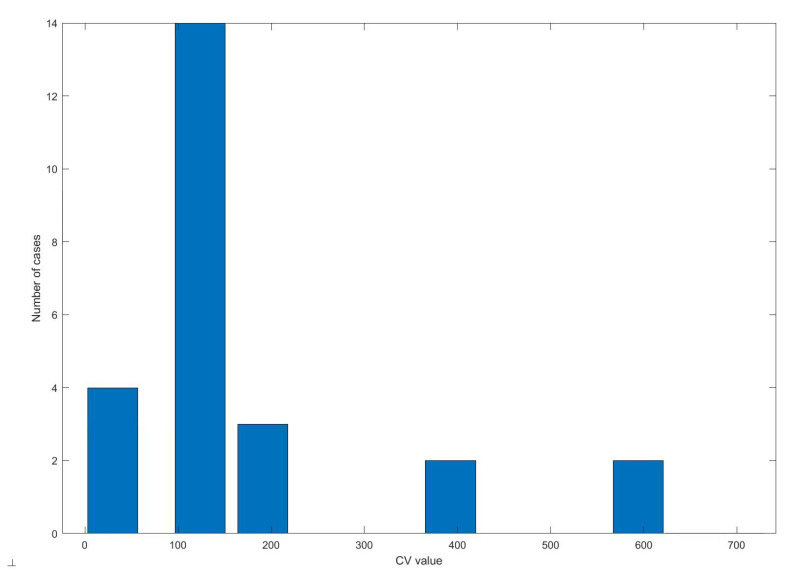
Histogram of coefficient of variation (CV) values.

Based on the estimated CV values, the features that showed variability below 50%, i.e., the number of GSR responses per minute (rpm), the number of significant GSRs and their energy, and the number of signal intersections with the regression line were rejected (Figure 6).

Because of the high number of available indicators, we decided to reduce the features again based on the value of the WCSS (Within-Cluster-Sum-of-Squares) parameter calculated according to Formula (3), as a sum of the squared difference between the central value of a given cluster ($Y_{i}$) and the $X_{i}$ observation—the next value of a given variable for all patients [56]. The WCSS value was calculated assuming the existence of two classes in the data set.

(3) $$WCSS=\sum {\left(X_{i}-Y_{i}\right)}^{2}$$

**Figure 7:**
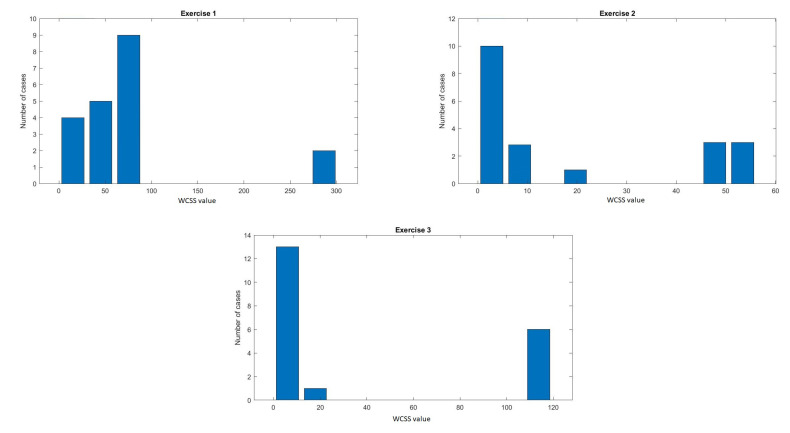
Histogram of Within-Cluster-Sum-of-Squares (WCSS) values.

This method was used to determine WCSS for each variable independently for fragments of signals from individual exercises and for the entire signal collected (Exercises 1–3). The threshold was selected based on the histogram analysis of the obtained results (Figure 7).

Sets of features for particular activities were used for the classification of the participants: Numbered lists can be added as follows:

1. Exercise 1—standard deviation, coefficient of the slope of the regression line, number of GSRs, value of the total signal sum,
2. Exercise 2—standard deviation, quartile deviation, coefficient of the slope of the regression line, GSR energy, minimum value of the signal, the 4th and 5th order moment, skewness, root of the mean square error, entropy,
3. Exercise 3—standard deviation, quartile deviation, coefficient of the slope of the regression line, number and energy of GSR, a minimum value of the signal, 4th and 5th order moment, skewness, kurtosis, the root of the mean square error, entropy, and energy of signal.

#### 4.3.2. Data Clustering

The data set prepared using this method was subjected to machine learning in several unsupervised learning variants. Due to the specific nature of psychological data, the data obtained from the EDA signal and the JAWS survey were divided into two classes each time.

**Figure 8:**
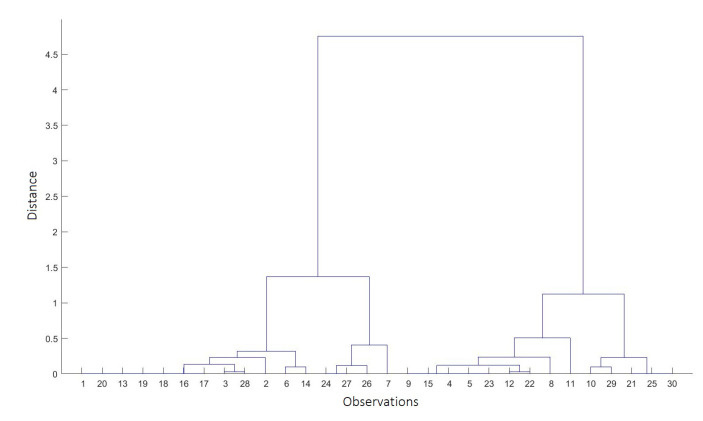
Dendrogram presenting the cluster hierarchy.

The first method of data division was an agglomerative hierarchical cluster tree [57] using different metrics of the distance between pairs of observation: Euclidean, Spearman’s rank correlation coefficient, and the correlation calculated as the difference between the maximum correlation and the value of the local correlation between two consecutive points. The clustering algorithm formed data clusters based on similarities between observations. During the first iteration, each observation formed a single-element cluster, and in the next step, the two clusters closest to each other were merged, thus creating an agglomeration. In this type of clustering, the algorithm moved up the hierarchy until it divided the data into the required number of clusters (Figure 8).

In the group of non-hierarchical methods, we used the k-means algorithm [58]. The method modifies the belonging of individual observations to classes until the variability within each group is optimised: the intraclass variability should be minimised whereas the interclass variability should be maximised.

The same clustering methods were applied to both the estimated features of the EDA signal and psychological data (general JAWS, JAWS_pos, JAWS_neg). While clustering, different approaches were tested. Each time, the results obtained from the clustering algorithm for the data collected from the JAWS questionnaire were used as reference values (Table 1).

#### 4.3.3. Clustering Optimisation

Two approaches to optimise the classifiers were proposed: feature space transformation using PCA, and feature space reduction by the contextual division of the feature set into groups and checking the quality of classification for each set independently.

Partial component analysis (PCA) is an operation of feature space transformation into a new space based on a linear combination of input features [59]. The transformation is based on the analysis of the main components of the input space and is used to reduce the number of variables and maximise their variability. The number of components in the new feature space is less or equal to the number of initial variables. Using PCA, the input data were converted to a new feature space while maintaining the number of variables.

The second optimisation approach was a contextual approach for signal features. Three sets of EDA signal features were selected (from the previously indicated):

1. Statistical set: standard deviation, quartile deviation, skewness, kurtosis, coefficient of the slope of the regression line,
2. Signal set: number of GSRs, energy of GSR, minimum, total sum,
3. Error set: 4th order moment, 5th order moment, rms, entropy.

### 4.4. Psychological Data Compliance

In order to verify the classification of the psychological data, the results of the k-means classifier were compared with the division made by an independent expert psychologist in terms of generally experienced emotions (JAWS), positive emotions (JAWS_pos), and negative emotions (JAWS_neg).

## 5. Results

### 5.1. Psychological Test

The following table (Table 2) presents the results of the JAWS test in terms of general evaluation of emotions, positive emotions, and negative emotions.

The mean for the results in the full version of the test for the entire group was 44.39 (SD = 5.25). Of all the participants, 71% obtained results equal to and higher than the mean for the group, which indicates a high frequency of experiencing both positive and negative emotions. On the scale of positive emotions, the mean for the group was 18.29 (SD = 5.51), whereas for negative emotions, this was 9.90 (SD = 3.51). Positive emotions above the mean for the group were reported by 54% of the participants, whereas 49% of them experienced negative emotions. An increased level of positive emotions is a state indicating high energy and a sense of involvement and pride in the achievements. The reduced level of positive emotions is characterised by uncertainty, discouragement, and sluggishness. An increased score on the subscale of negative emotions indicates a state of distress and experiencing many emotions simultaneously, and is connected with aversive states, i.e., anger, nervousness, and resentment. Low scores in terms of negative emotions are associated with experiencing quiet and positivity in the assessment of the person himself or herself and the surrounding world.

### 5.2. Data Classification

The results of the subsequent stages of processing are presented below. Each time, the results obtained from the clustering algorithm for the data collected from the JAWS questionnaire for individual emotions were used as reference values.

#### 5.2.1. Feature Reduction

The CV values were varied. Based on the analysis of the histogram, the differential threshold was set at the level of 50% (Figure 6). The estimated WCSS values ranged from 0.02 to 300. Based on the histogram, the threshold was determined for fragments of signals from individual exercises and for the entire signal collected. Of 25 features, thirteen most differentiating were chosen. Table 3 shows the means of values (WCSS) for features of the EDA signal recorded during individual exercises.

The above data were characterised by the greatest variability between individual observations. Each of them showed the WCSS coefficient lower than the threshold. Some differences were observed while analysing the individual values obtained for exercises. During exercises, signal tonicity was characterised by an upward trend, with slight differences between individual stages. However, the mean number of galvanic skin responses changed: it was the greatest during the first exercise (five times higher than for exercise two and three), but their energy was not the highest. This means that at this stage of the test GSRs were frequent but not very strong. Positive results of the kurtosis coefficient for all stages of the study indicate concentration of results around the mean value. The value of entropy was calculated according to Shannon’s definition as the smallest average amount of information needed to encode an event with a given probability [60]. The highest value was again obtained for the first exercise.

#### 5.2.2. Clustering and Optimisation

The results of patient clustering based on physiological and psychological data collected from JAWS questionnaire were compared both with and without the use of a PCA algorithm.

Table 4 shows the percentage accuracy of clustering (ACC) using individual methods of EDA features compared to JAWS results, using the approach of a general assessment of emotions. Each time, the results obtained from the classification algorithm for the data collected from the JAWS questionnaire were used as reference values.

It can be observed based on the calculations that each time the PCA transformation was used, it improved the accuracy of the classifier. The best results for all three exercises were obtained using the k-means classifier, both with and without PCA. This classifier turned out to be most accurate for Exercise 3.

Based on the above results, the basic characteristics, i.e., sensitivity (TPR, True Positive Rate) and specificity (TNR, True Negative Rate) were calculated for the most accurate classifier (k-means) according to the following equations:

(4) $$TPR=\frac{True\;positive}{True\;positive+False\;negative}$$

(5) $$TNR=\frac{True\;negative}{True\;negative+False\;positive}$$

True Positive values refer to observations classified as positive by the classifier based on JAWS and EDA coefficients. False Negative means positive observations that are classified as negative. Furthermore, True Negative refers to the samples classified in both cases as a negative class, whereas False Positive means negative samples but classified as a positive class. The table below (Table 5) presents the sensitivity and specificity of the k-means classifier during different stages of the test.

The k-means classifier for the data from Exercise 3 was the most accurate. It is presumed that sensitivity and specificity at the level of 80% may indicate a consistent classification of the majority of observations coming from physiological data compared to psychological data. Therefore, in the following stages of the analysis, we decided to further analyse data only from Activity 3.

As mentioned before, the psychological evaluation of the JAWS test allows for dividing people according to general emotions, but also separately for JAWS_pos and JAWS_neg. So far, the results of the classification have been based on the general evaluation of the psychological questionnaire and EDA signal features. Table 6 below shows the accuracy, sensitivity, and specificity of the k-means classifier compared to an independent evaluation of positive and negative emotions.

The above analysis reveals that the highest accuracy of the classifier occurred for the comparison of participants’ grouping based on EDA signal features with negative emotions.

The accuracy of the classification of physiological data was also verified based on the sets of features, according to the previously proposed division into three sets (Table 7).

Again it was shown that the highest grouping accuracy occurred for the combination of EDA data with JAWS_neg. However, not all the sets of features were characterised by the expected results. The smallest convergence was obtained for a set of statistical parameters. Combined with PCA, characteristic signal parameters (Set 2) and errors (Set 3) allowed for correct classification at an average level of 81.71%.

### 5.3. Psychological Data Compliance

Table 8 shows the convergence of the division of the participants into two groups by comparison of the k-means classifier for Exercise 3 and the independent evaluation of the psychologist, in terms of emotions experienced (JAWS), and separately for positive emotions (JAWS_pos) and negative emotions (JAWS_neg).

The k-means classifier reached maximum accuracy of classification, with convergence at the level of 78.05% with independent division made by the psychologist in the case of division of participants in terms of negative emotions (JAWS_neg).

## 6. Discussion

The present study attempted to select a methodology and an optimal set of physiological data that allow for objectivisation of psychological tests to support experts in the effective assessment of the patient’s emotional state during physiotherapeutic procedures.

The presented protocol was based on the acquisition of the electrodermal activity signal and a questionnaire used to evaluate the intensity of JAWS emotions. The advantage of using the psychological test was to obtain information about both negative and positive emotions since optimal coping with stress involves a skilful combination of negative and positive emotions [7].

The set of physiological and psychological data was subjected to a process of machine learning with optimization. During testing of different classification methods, the use of PCA transformation improved each time the accuracy of the classifier. The best results were obtained for the k-means classifier during Exercise 3, without (70.73%) and with PCA (80.49%). Sensitivity and specificity of this classifier were 80.64% and 80%, respectively, which indicates a consistent classification of the majority of observations coming from physiological compared to psychological data. In medical research, sensitivity and specificity are extremely important indicators, especially in the case of screening [61]. In the approach presented here, both sensitivity and specificity are considered important because the subjects are not classified as healthy or ill, but are divided into classes according to the dominant positive or negative emotions experienced during exercises. It was found during the independent evaluation of positive emotions (JAWS_pos) and negative emotions (JAWS_neg) that the highest accuracy of the classifier occurred for the comparison of participants’ grouping based on EDA signal coefficients with negative emotions (80.48%). In the contextual division of the feature set, the classifier was again most precise for negative emotions combined with the signal parameter set (78.05%) and error parameter set (85.37%). The results are consistent with behavioural and physiological studies, because a person experiences negative emotions more strongly than positive ones, and therefore it is easier to correlate EDA features with JAWS_neg [34]. Comparison of the accuracy of the k-means classification with the independent division made by a psychologist revealed again the best results for negative emotions (78.05%). This is not the result that was expected to be obtained, as the high emotionality of the participants makes interpretation difficult. Furthermore, the advantage of the mean value in terms of positive emotions over negative emotions may indicate that the negative effect was unimportant for the exercise. The critical positivity ratio (the Losada ratio), which did not exceed the indicated level of 2.9 and amounted to 1.8, does not mean that the emotions experienced lead to expanding resources, i.e., development and increasing awareness.

To the best of our knowledge, no studies have examined the patient’s emotional state during rehabilitation. Studies in the literature have reported only related methods of emotional analysis using external devices and machine learning algorithms. In their study, TaheriNejad et al. also studied emotions by recording physiological signals using an Empatica E4 device [33]. A very important difference should be emphasised: in the quoted paper, emotions were fully controlled by the presentation of videos dedicated to evoking specific feelings rather than spontaneous reactions to the stimuli, and subsequent psychological analysis, as in the case of the protocol presented in this study. The paper by Liu et al. proposed a method of recognising emotions, which was also based on machine learning using Support Vector Machine and EDA signal [34]. They obtained 83.57% accuracy on learning data and 66.67% on test data, which shows comparable accuracy of classification (85.37%). However, emotions were again assessed in a subjective manner, and therefore the study lacked reference to the actual feelings experienced by the participants. Gjoreski et al. used an emotion detection algorithm based on short-term psychological stress and obtained the detection accuracy of 70% for stress events at 95% accuracy [27].

All the above reports lead to the conclusion that the use of EDA signal features to analyse emotions and support psychological evaluation is fully justified. The presented literature reports the results of the classification of patient’s emotions based only on the assessment of events by other people (most often patients watched videos), who assessed that a given situation may cause a given emotion. Unfortunately, this approach is often wrong, because every person experiences different situations differently and it is impossible to assess the feelings independently. Therefore, in the approach used in the present paper, physiological data were compared with psychological data derived directly from the patient, and, based on the classification, the results were obtained at an equally high level of accuracy.

## 7. Summary

Based on the above analysis, the set of analytical parameters that can be proposed for future research represents a group of coefficients of various types of signal errors. It provides satisfactory results both in the approach of a general analysis of emotions and in the division into positive and negative emotions. Based on the analysis and literature review, negative emotions are more important from the standpoint of the therapy, because such people may be less involved, be more problematic, and unwilling to cooperate with the therapist. Correlation of physiological data with psychological sources will be under further investigation of our research.

## Figures and Tables

**Table 1:** Distribution of psychological data within clusters.

	Threshold	Range	Cluster 1	Cluster 2
JAWS	44.5	12–60	Positive (*n* = 21)	Negative (m = 20)
JAWS_pos	18.5	6–30	Positive (*n* = 19)	Neutral (m = 22)
JAWS_neg	10	6–30	Negative (*n* = 15)	Neutral (m = 26)

Note: n, m—number of cases.

**Table 2:** Descriptive statistics of psychological data.

Variable	JAWS	JAWS_poz	JAWS_neg
Mean	44.39	18.29	9.90
Standard deviation	5.25	5.51	3.51
Median	45	18	9
Min	33	6	6
Max	57	27	21
Possible range	12–60	6–30	6-30

Note: *N* = 41. JAWS—global scale, JAWS_pos—scale for positive emotions; JAWS_neg—scale for negative emotions.

**Table 3:** Mean values of features during activity (by WCSS).

Feature	Exercise 1	Exercise 2	Exercise 3
Standard deviation	0.232	0.103	0.102
Quartile deviation	0.341	0.184	0.167
Coefficient of the slope of the regression line	0.002	0.005	0.004
Number of GSRs	7.634	1.683	1.341
Energy of GSR	3.898	4.482	4.513
Minimum value	3.510	4.563	4.972
4th order moment	1.348	0.044	0.047
5th order moment	2.739	−0.004	0.043
Skewness	0.303	0.206	0.129
Kurtosis	3.256	4.707	5.109
Root mean square	3.885	4.707	5.109
Entropy	0.952	0.426	0.502

**Table 4:** Comparison of the effect of classifiers.

	Exercise 1		Exercise 2		Exercise 3	
**Classifier**	**without PCA** **[%]**	**with PCA** **[%]**	**without PCA** **[%]**	**with PCA** **[%]**	**without PCA** **[%]**	**with PCA** **[%]**
Cluster tree Euclidean	51.22	60.98	39.02	60.98	39.02	60.98
Cluster tree Spearman	31.71	36.59	51.22	51.22	58.54	41.46
Cluster tree Correlation	19.51	36.59	21.95	36.59	48.29	48.78
**K-mean**	**41.46**	**68.29**	**68.29**	**70.73**	**70.73**	**80.49**

**Table 5:** Evaluation of the k-means classifier; accuracy (ACC), sensitivity (TPR), and specificity (TNR).

	Exercise 1		Exercise 2		Exercise 3	
	**without PCA** **[%]**	**with PCA** **[%]**	**without PCA** **[%]**	**with PCA** **[%]**	**without PCA** **[%]**	**with PCA** **[%]**
ACC	41.46	68.29	68.29	70.73	**70.73**	**80.49**
TPR	80.00	96.29	88.89	70.83	**70.83**	**80.64**
TNR	12.50	14.29	35.71	64.71	**73.33**	**80.00**

**Table 6:** Evaluation of the k-means classifier for separate Job-related Affective Well-being Scale (JAWS) evaluation.

	Exercise 3 + JAWS_pos		Exercise 3 + JAWS_neg	
	**without PCA** **[%]**	**with PCA** **[%]**	**without PCA** **[%]**	**with PCA** **[%]**
ACC	60.98	78.05	60.98	**80.49**
TPR	75.00	92.00	78.95	**95.45**
TNR	30.77	56.25	45.45	**63.13**

**Table 7:** Evaluation of the k-means classifier for individual sets of features.

Feature Set		EDA+JAWS		EDA+JAWS_pos		EDA+JAWS_neg	
	Coefficient	without PCA [%]	with PCA [%]	without PCA [%]	with PCA [%]	without PCA [%]	with PCA [%]
set 1	ACC	63.41	73.17	53.65	78.05	46.34	68.29
	TPR	78.26	91.67	57.14	84.62	48.00	91.30
	TNR	44.44	52.94	50.00	66.67	43.75	38.89
set 2	ACC	58.53	68.29	51.22	78.05	60.98	**78.05**
	TPR	76.19	84	70.00	88.89	64.71	**81.15**
	TNR	31.58	43.75	45.16	68.56	58.33	**71.43**
set 3	ACC	78.05	**85.37**	58.54	68.29	**78.05**	**85.37**
	TPR	96.30	**95.45**	66.67	77.78	**91.30**	**88.46**
	TNR	42.86	**73.68**	42.11	66.67	**68.75**	**80.00**

**Table 8:** Comparison of the division of the participants according to the classifier and performed by expert psychologist.

	JAWS		JAWS_pos		JAWS_neg	
	**without** **PCA [%]**	**with PCA** **[%]**	**without** **PCA [%]**	**with PCA** **[%]**	**without** **PCA [%]**	**with PCA** **[%]**
ACC	65.85	73.17	60.98	73.17	63.41	**78.05**
